# Sunlight and Health

**Published:** 1846-02

**Authors:** 


*Sunlight and Health.*—[Chambers' Edinburgh Journal abounds with admirable papers, occasionally on matters of essential importance to mankind would they heed the admonitions. Read this.]

Turning now to animal economy, we find growth, health, and developement also curiously affected by the absence or presence of the solar influence. Dr. Edwards has shown that if tadpoles be nourished with proper food, and exposed to the constantly renewed action of the water, (so that their touched respiration may be maintained,) but are entirely deprived of light, their growth continues, but their metamorphosis into air-breathing animals is arrested, and they remain in the form of large tadpoles. He also observes that persons who live in caves or cellars, or in very dark and narrow streets, are apt to produce deformed children; and that men who work in mines are liable to disease and deformity beyond what the simple closeness of the atmosphere would be likely to produce. It has been stated, on the authority of A. Wylie, that the cases of disease, on the dark side of an extensive barrack at St. Petersburgh, have been uniformly for many years in the proportion of three to one on the side exposed to strong light. Further, Dupuytren relates the case of a lady whose maladies had baffled the skill of several eminent practitioners. The lady resided in a dark room (on which the sun never shone) in one of the narrow streets of Paris. After a careful examination, Dupuytren was led to refer her complaints to absence of light, and recommended her removal to a more exposed situation. This change was followed by the most beneficial results; all her complaints vanished. The more, therefore, that animals are exposed to the influence of light, the more free are they in ordinary circumstances, from irregular action and deformity.

[A Richmond paper, in extracting and commenting on this, thus remarks:]

In another part of the article, it is shown that heat and light alone, without the solar radiation, will not suffice for the health of vegetables or of animals; else the artificial fires and lustres of our apartments would have that effect; but they do not. An indispensable agent is actinism.

Now, do not the foregoing facts prove the unhealthiness of changing night into day, as many of our fashionable and semi-fashionable, studious and psuedo-studious people do? The unhealthiness of wasting in bed the bright and bracing hours of early morning, when nature bids us be out of doors digging, or walking, or riding? Is not the balefulness of dark rooms made palpable? Draw aside those curtains—open those window-blinds, thou sluggard, and let Aurora and the sun, looking full into thy chamber, shame thee forth, if they cannot charm thee forth, to inhale strength and health in those best and most beauteous hours of the day.—*Bost. Med. and Surg. Journal.*

